# Characterization of an iPSC-based barrier model for blood-brain barrier investigations using the SBAD0201 stem cell line

**DOI:** 10.1186/s12987-023-00501-9

**Published:** 2023-12-19

**Authors:** Burak Ozgür, Elena Puris, Andreas Brachner, Antje Appelt-Menzel, Sabrina Oerter, Viktor Balzer, Mikkel Roland Holst, Rasmus Folmann Christiansen, Kathrine Hyldig, Stephen T. Buckley, Mie Kristensen, Seppo Auriola, Allan Jensen, Gert Fricker, Morten Schallburg Nielsen, Winfried Neuhaus, Birger Brodin

**Affiliations:** 1 Department of Pharmacy, University of Copenhagen, Universitetsparken 2, Copenhagen, DK-2100 Denmark; 2 Biotherapeutic Discovery, H. Lundbeck A/S, Valby, DK-2500 Denmark; 3 Institute of Pharmacy and Molecular Biotechnology, Ruprecht-Karls-University, Heidelberg, Germany; 4 AIT - Austrian Institute of Technology GmbH, Vienna, 1210 Austria; 5 Chair Tissue Engineering and Regenerative Medicine (TERM), University Hospital Würzburg, 97070 Würzburg, Germany; 6 Fraunhofer Institute for Silicate Research ISC, Translational Center Regenerative Therapies (TLC-RT) Röntgenring 11, 97070 Würzburg, Germany; 7 Department of Biomedicine, Aarhus University, Aarhus, DK-8000 Denmark; 8 Global Research Technologies, Novo Nordisk A/S, Måløv, DK-2760 Denmark; 9 School of Pharmacy, University of Eastern Finland, Kuopio, Finland; 10 Department of Medicine, Faculty of Medicine and Dentistry, Danube Private University, Krems, 3500 Austria

**Keywords:** Human induced pluripotent stem cells (hiPSCs), Brain capillary endothelial-like cells (BCECs), Blood-brain barrier (BBB), Tight junctions, Solute carriers (SLC) transporters, Efflux transporters

## Abstract

**Background:**

Blood-brain barrier (BBB) models based on primary murine, bovine, and porcine brain capillary endothelial cell cultures have long been regarded as robust models with appropriate properties to examine the functional transport of small molecules. However, species differences sometimes complicate translating results from these models to human settings. During the last decade, brain capillary endothelial-like cells (BCECs) have been generated from stem cell sources to model the human BBB in vitro. The aim of the present study was to establish and characterize a human BBB model using human induced pluripotent stem cell (hiPSC)-derived BCECs from the hIPSC line SBAD0201.

**Methods:**

The model was evaluated using transcriptomics, proteomics, immunocytochemistry, transendothelial electrical resistance (TEER) measurements, and, finally, transport assays to assess the functionality of selected transporters and receptor (GLUT-1, LAT-1, P-gp and LRP-1).

**Results:**

The resulting BBB model displayed an average TEER of 5474 ± 167 Ω·cm^2^ and cell monolayer formation with claudin-5, ZO-1, and occludin expression in the tight junction zones. The cell monolayers expressed the typical BBB markers VE-cadherin, VWF, and PECAM-1. Transcriptomics and quantitative targeted absolute proteomics analyses revealed that solute carrier (SLC) transporters were found in high abundance, while the expression of efflux transporters was relatively low. Transport assays using GLUT-1, LAT-1, and LRP-1 substrates and inhibitors confirmed the functional activities of these transporters and receptors in the model. A transport assay suggested that P-gp was not functionally expressed in the model, albeit antibody staining revealed that P-gp was localized at the luminal membrane.

**Conclusions:**

In conclusion, the novel SBAD0201-derived BBB model formed tight monolayers and was proven useful for studies investigating GLUT-1, LAT-1, and LRP-1 mediated transport across the BBB. However, the model did not express functional P-gp and thus is not suitable for the performance of drug efflux P-gp reletated studies.

**Supplementary Information:**

The online version contains supplementary material available at 10.1186/s12987-023-00501-9.

## Introduction

The blood-brain barrier (BBB) is a function of the neurovascular unit that contributes to the strict regulation of the influx and efflux of substances between the blood to the brain parenchyma [1]. The brain capillaries provide an interface for blood-brain exchange with an estimated surface area of 12–18 m^2^ in human adults with short diffusion distances from capillaries to neurons [2]. However, the physical tightness of the brain endothelium, along with the expression of a range of efflux transporters and solute carriers (SLCs), as well as low pinocytic activity, and the presence of metabolic enzymes together restrict the entry of a large number of small molecule drugs and biotherapeutics [3, 4]. Thus, the BBB represents a major obstacle when developing new medicines with CNS targets.

The low paracellular permeability, and thus physical tightness, of the BBB is primarily due to intercellular tight junction proteins, including claudins (CLDNs), occludin (OCLN), and junctional adhesion molecules (JAMs) [5], connecting the endothelial cells. The tight junction proteins are closely associated with the scaffolding and regulatory proteins zonula occludens (ZO)-1-3, the adherens junction VE-cadherin, and PECAM-1 [6].

Several in vitro BBB models where brain capillary endothelial cells are grown in monolayers on permeable supports (either in mono-culture or co-culture with astrocytes and pericytes) have been developed in order to facilitate screening of drug transport across the BBB (for review, see [7]). The sources of brain capillary endothelial cells are often animal brain tissues, such as pigs, calves, and rodents. The models display important BBB properties by expressing tight junction proteins, SLCs, receptor systems, and efflux transporters [7–9]. However, these models are labor-intensive, which limits their use for high throughput screening purposes in drug development studies [10]. Another limitation of these models is that they are based on animal-derived brain tissue and thus exhibit species-species differences compared to the human BBB. Immortalized cell lines of human origin or primary cells obtained from human brains are other often-used approaches for establishing human BBB models [11–15]. However, the immortalized endothelial cell lines tend to express relatively low levels of tight junctions, resulting in leakier barriers, making them suboptimal for studies on drug transport pathways across the BBB [7]. Further, the few models using primary brain capillary endothelial cells from human brains are often obtained from biopsies (diseased brain), autopsies, or commercial vendors, which only have limited information on the source [13, 14]. The last decade has witnessed the establishment of several in vitro BBB models based on human stem cell sources. The in vitro BBB models include the use of human induced pluripotent stem cells (hiPSCs), umbilical cord blood-derived hematopoietic stem cells, and amniotic fluid-derived stem cells [16–22]. The stem cells are cultured in complex media to differentiate them into brain capillary endothelial-like cells, which are subsequently cultured on permeable supports to form cell monolayers [16, 17, 19, 21, 23]. The resulting cell monolayers exhibit restrictive barrier properties and expression of essential proteins, such as tight junctions, solute carriers, receptors, and efflux transporters [24]. Therefore, these hiPSC-derived models represent a valuable tool for mechanistic drug transport studies across the BBB. Moreover, hiPSCs offer the possibility to model a patient-specific BBB model by obtaining cell sources from patients with diseased genetic backgrounds [25, 26].

The present study aimed at establishing a BBB model using the hiPSC line SBAD0201 and at characterizing the BCECs differentiated thereof as well as at investigating the resulting BBB transwell models on tissue-specific transport phenomena. The obtained model was characterized by paracellular tightness, expression of tight junctions, adherens junctions, SLC-transporters, receptors, and efflux transporter expression, as well as its function as a tool for assessing BBB permeation of CNS drug candidates.

## Materials and methods

All chemicals and reagents were obtained from Sigma-Aldrich (Broendby, Denmark) unless otherwise stated. The human induced pluripotent stem cell (hiPSC) line SBAD0201 was obtained by reprogramming fibroblasts obtained from donated human skin tissue by IMI StemBANCC, and the cell line was kindly provided by Dr Zameel Cader, University of Oxford. Human cerebral microvascular endothelial cells (hCMEC/D3) cells were kindly by Prof. Dr. Jörg Huwyler (University of Basel, Switzerland),

### Cell culture

SBAD0201 cells were maintained on Matrigel-coated 6-well plates in mTeSR1 medium at 37 °C, 5% CO_2_, and 95% atmospheric air and passaged with Versene (for maintenance) or Accutase (for differentiation) at 80% confluency. SBAD0201 cells were differentiated into BCECs using the protocol established by Lippmann et al. with some modifications [16, 17]. The differentiation was initiated by seeding 7.5 × 10^4^ cells pr well onto Matrigel-coated 6-well plates. The cells were maintained in mTesR1 for three days (from Day − 3 to Day 0). On Day 0, the medium was changed to an unconditioned medium (UM) consisting of DMEM/F12, 1x MEM non-essential amino acids (NEAA), 1 mM L-glutamine, 0.1 mM β-Mercaptoethanol, and 20% knockout serum replacement. The cells were cultured in UM for six days with a medium change daily (from Day 0 to Day 6). The medium was changed to endothelial cell medium +/+ (hESFM, 0.5% B27, 20 ng/mL bfGF, 10 µM retinoic acid (RA)), abbreviated EC+/+, and the cells were expanded in this medium for two days. On Day 8, the cells were dissociated from the culture plate using accutase (25 min at 37 °C). The cells were subsequently seeded onto collagen IV-coated (400 µg/mL) and fibronectin-coated (100 µg/mL) polyester transwell inserts (Greiner Bio-One #665,641, 1.12 cm^2^, pore size 0.4 μm) (1 × 10^6^ cells/cm^2^) in a 12-well Transwell® plate. On day 9, the medium was changed to EC without bfGF and RA (EC-/-). The cells were ready for experimental use on Day 10.

hCMEC/D3 cells were cultured in endothelial cell growth medium 2 (PromoCell GmbH, Heidelberg, Germany) supplemented with 2% (v/v) fetal calf serum (FCS), 5 ng/mL recombinant human epidermal growth factor (EGF), 10 ng/mL recombinant human basic fibroblast growth factor (bFGF), 20 ng/mL insulin-like growth factor (IGF-1), 0.5 ng/mL recombinant human vascular endothelial growth factor 165, 1 µg/mL ascorbic acid, 22.5 µg/mL heparin, 0.2 µg/mL hydrocortisone, and 1% penicillin–streptomycin. The cells were maintained in pre-coated (rat tail collagen type I) T-75 flasks (Corning, USA) at a seeding density of 100,000 cells/10 mL under standard conditions for a period of 7 days. The cell culture medium was refreshed every 2 to 3 days.

### Isolation of mRNA and cDNA synthesis

Total RNA was isolated from the cells on Day 0, Day 6, Day 8, and Day 10 (experimental day), and for each passage, at least lysates of three permeable supports were pooled together to generate one RNA sample. The mRNA was extracted from the cell culture and isolated using the NucleoSpin RNA Kit according to the manufacturer’s instructions (Machery-Nagel, Düren, Germany). The concentration and purity of the RNA were measured using a NanoDrop 2000c spectrophotometer (Thermo Fisher Scientific). Reverse transcription was performed with 250 ng of RNA per reaction using the High-Capacity cDNA Reverse Transcription kit (Applied Biosystems, Naerum, Denmark) according to the manufacturer’s instructions. The reverse transcription was performed in a PTC-200 Thermal Cycler (MJ Research, Quebec, Canada). The real-time quantitative PCR analysis was performed using Fluidigm Biomark platform (using 96 samples x 96 target chips as described previously [27–30]. All cDNA samples were pre-amplified prior to analysis using the 96 × 96 high throughput. A reaction mixture consisting of cDNA, primers (1 µM of each forward and reverse primer), water (PCR-grade), and the master mix was used. Primers were validated by controlling the melting curves, efficiencies, and, lastly the product sizes using agarose gels. Cycle conditions were as follows: Pre-incubation at 95 °C for 20 s, followed by 40 cycles at 95 °C for 3 s and 60 °C for 30 s, and melting at 95 °C for 15 s, 60 °C for 1 min, and 95 °C for 15 s. The efficiencies of the primer pairs were determined in-house by estimating the slope from a serial dilution calibration curve using the equation E = 10^(−1/slope)^. A set of two reference genes (*B-actin and GAPDH*) was used to normalize the 2^−ΔCt^-values.

### Absolute quantification of transporter protein expression

The absolute protein amounts of following membrane transporters: ABCB1, ABCG2, ABCC1, ABCC4, SLC2A1, SLC7A1, SLC7A5, SLC3A2, SLC16A1 and SLC27A1 were quantified in crude membrane fractions of undifferentiated SBAD0201 cells and SBAD0201 derived endothelial-like cells, and lastly in hCMEC/D3 cells. First, crude membrane factions were isolated from the cells using ProteoExtract® Subcellular Proteome Extraction Kit (Merck KGaA, Darmstadt, Germany) according to the manufacturer`s instructions. Total protein concentrations were measured in the samples by the Lowry method using the Bio-Rad DC protein assay (Bio-Rad, Hercules, CA). The preparation of peptide samples and Liquid chromatography with tandem mass spectrometry (LC-MS/MS) -based quantitative targeted absolute proteomic analysis were performed as described previously [31–34]. Briefly, aliquots of the samples containing 50 µg of total protein were solubilized in 7 M guanidine hydrochloride, 500 mM Tris–HCl (pH 8.5), and 10 mM EDTA. Subsequently, total proteins were reduced after adding dithiothreitol followed by *S*-carbamoylmethylation with iodoacetamide. The proteins were precipitated after adding methanol and chloroform and subsequently dissolved in 6 M urea in 0.1 M Tris–HCl (pH 8.5). The samples were diluted by adding 0.1 M Tris–HCl (pH 8.5) spiked with a mixture of stable-isotope labelled quantified peptides (JPT Peptide Technologies GmbH, Berlin, Germany). Lys-C and Protease-Max (Promega, Madison, WI, USA) were added to the samples, which were then incubated at room temperature for 3 h. Tosylphenylalanyl chloromethyl ketone-treated trypsin (Promega, Madison, WI, USA) was used for digestion with enzyme to substrate ratio of 1:100 at 37 °C for 16 h. Afterwards, the samples were mixed with formic acid in water 20% (v/v) and centrifugated at 14,000 × g for 5 min at 4 °C. The resulting supernatants were injected the Agilent 1290 Infinity LC (Agilent Technologies, Waldbronn, Germany) system, which was connected to an Agilent 6495 Triple Quadrupole Mass Spectrometer equipped with an ESI source (Agilent Technologies, Palo Alto, CA, USA). The separation and detection of target peptides (Appendix 1) was performed using an Advance Bio Peptide Map column (2.1 × 250 mm, 2.7 μm) and positive ion multiple reaction monitoring (MRM) mode as previously described [35, 36]. The absolute quantitation of the target proteins was done based on a unique peptide (Table S1), which was selected according to the *in silico* peptide selection criteria [33, 35–38]. Data were obtained using the Agilent MassHunter Workstation Acquisition software (Agilent Technologies, Data Acquisition for Triple Quad., version B.03.01), and data procession was conducted with Skyline software (version 4.1). The absolute protein expression levels and the limit of quantification were calculated as reported in our previous studies [31, 32].

### Immunostaining

SBAD0201-derived BCECs were fixed on the experimental day with 3% (v/v) paraformaldehyde in PBS for 10 min and subsequently permeabilized in 0.1% (v/v) Triton™ X-100 in PBS for 5 min at room temperature. The samples were blocked in 2% (w/v) bovine serum albumin (BSA) in PBS for 30 min at room temperature. The samples were incubated overnight at 4 °C with the antibodies listed in Table 1. This was followed by three washing steps with PBS + 2% (w/v) BSA for 5 min each. The samples were incubated for 30 min at room temperature with Alexa 488-conjugated secondary antibody (diluted 1:200), goat anti-rabbit or anti-mouse IgG (Molecular Probes, Leiden, the Netherlands) and combined with DAPI (1.5 µM, Molecular Probes, Leiden, the Netherlands) for visualizing the cell nuclei. Next, the samples were washed twice in ice-cold PBS and mounted on coverslips. The samples were visualized using a confocal laser scanning microscope (plan-apochromat 63x/NA 1.4, Zeiss LSM 710, Carl Zeiss, Jena, Germany).

**Table 1:** Overview of antibodies used in the present study

Target protein	Clonality	Host	Dilution factor	Supplier and catalogue ID
Primary antibodies				
VE-Cadherin	Polyclonal	Rabbit	1:100	Lundbeck A/S
PECAM-1	Polyclonal	Mouse	1:100	Lundbeck A/S
Von Willebrand Factor	Polyclonal	Rabbit	1:100	Lundbeck A/S
Occludin	Polyclonal	Rabbit	1:100	Lundbeck A/S
Claudin-5	Polyclonal	Rabbit	1:100	Lundbeck A/S
Zonula occludens-1	Polyclonal	Rabbit	1:100	Lundbeck A/S
Glut-1	Polyclonal	Rabbit	1:100	Lundbeck A/S
LAT-1	Polyclonal	Rabbit	1:100	Bioss Antibodies (bs-10125R)
P-glycoprotein	Polyclonal	Rabbit	1:100	Lundbeck A/S
LRP-1	Monoclonal	Rabbit	1:100	Abcam (Ab92544)
Secondary antibodies conjugated with Alexa-488 or HRP				
Anti-rabbit	Polyclonal	Goat	1:200	Life Technologies (AV1008)
Anti-mouse	Polyclonal	Goat	1:200	Invitrogen (AH017)

### Transport studies

Transport assays were performed on Day 10 in cell medium (EC-/-) or in HBSS supplemented with 10 mM HEPES (pH 7.4), 0.375% sodium carbonate, and 0.05% BSA (abbreviated HBSS+) after washing the cell monolayers twice with transport buffer (HBSS+). This was followed by an equilibration period in transport buffer for 15 min at 37 °C on a temperature-controlled shaking table (Unimax 2010 Shaker, Heidolph, Schwabach, Germany) with a circular horizontal rotation of 90 rpm. The volumes were 700 µL and 1500 µL in the apical and basolateral chambers, respectively. The transport was initiated by spiking the donor chamber with the radiolabeled compounds [^3^H]-propanolol, [^14^C]-mannitol, [^3^H]-glucose, [^3^H]-L-leucine, [^3^H]-digoxin, or [^125^I]-angiopep-2, or the fluorophores Natrium fluorescein (NaF), FITC-dextran (4 kDa), 6-NBDG, or rhodamine 123. Samples containing radio-labelled compounds were transferred to scintillation vials, and 2 mL of Ultima Gold scintillation fluid was added. The radioactivity was counted using a Tri-Carb 2910 TR Liquid Scintillation Analyzer. For the fluorophores, samples were directly pipetted into 96-well plates. The samples were analyzed with a microplate reader (NOVOstar Microplate Reader, BMG Labtech, Offenburg, Germany), where the excitation and emission wavelengths were set to 485 and 520 nm, respectively.

#### Paracellular and transcellular marker transport experiments

Bidirectional transport assays involving [^3^H]-propanolol (0.1 µCi·mL^− 1^), [^14^C]-mannitol (0.1 µCi·mL^− 1^), NaF (10 µM), and 4 kDA FITC-dextran (25 µM) were conducted in HBSS^+^ (after two washes in HBSS). The transport was examined for a time course of two hours, except with 4 kDa FITC-dextran, which was run for four hours. Aliquots were removed from the receiver chambers (50 µL from the apical or 100 µL from the basolateral compartment) after 15, 30, 45, 60, 90, and 120 min and 30, 60, 90, 120, 180 and 240 min for 4 kDa FITC-dextran. The volume of the withdrawn samples was replaced with pre-heated HBSS^+^ (37 °C).

#### Transport of GLUT-1 and LAT-1 substrates

The apical-to-basolateral (A-B) transendothelial transport of [^3^H]-glucose (1 µCi·mL^− 1^), 6-NBDG (100 µM), or [^3^H]-L-leucine (1 µCi·mL^− 1^) was evaluated across the SBAD0201-derived monolayers. HBSS without glucose was used for transport assays involving [^3^H]-glucose and 6-NBDG, whereas HBSS^+^ was used for [^3^H]-L-leucine. Aliquots were withdrawn from the basolateral chamber after 10, 20, 30, 40, 50, and 60 min, and the withdrawn volumes were replaced with pre-heated HBSS without glucose or HBSS^+^. The function of GLUT-1 and LAT-1 were evaluated by co-application of the GLUT-1 inhibitors BAY876 (1 and 10 µM) and phloretin (100 µM) and the LAT-1 inhibitors JPH203 (10 µM) and BCH (100 µM). The inhibitors were added to the transport buffer in both chambers 15 min before the addition of the substrates.

#### Transport of P-gp substrates

For bidirectional transport assays involving [^3^H]-digoxin (1 µCi·mL^− 1^) and rhodamine 123 (5 µM), the transport was conducted in EC-/-. On the experimental day, the cell medium was changed to fresh EC-/- two hours prior to the initiation of the transport assays. Aliquots were withdrawn from the receiver chambers (50 µL from the apical or 100 µL from the basolateral compartment) after 15, 30, 45, 60, 90, and 120 min. The volume of the withdrawn samples was replaced with pre-heated EC-/- (37 °C). The P-gp inhibitor zosuquidar (0.4 µM) was added to the culture medium in both chambers 15 min prior to the addition of [^3^H]-digoxin or rhodamine 123 to probe the functionality of P-gp. The cellular uptake of the substrates was probed after ended transport assays.

#### Transport of the LRP-1 ligand Angiopep2

Apical-to-basolateral (A-B) directional transendothelial transport of [^125^I]-angiopep2 (1 µCi·mL^− 1^) was conducted in HBSS^+^. Aliquots were withdrawn from the basolateral chamber after 30, 60, 90, 120, 180, and 240 min, and the withdrawn volumes were replaced with pre-heated HBSS^+^. The filter content of [^125^I]-angiopep2 was probed after the four hours transport assay. The functionality of LRP-1 was evaluated by co-application of 25 µM non-labeled angiopep-2, which was added simultaneously with the substrate.

### Data treatment and statistics

Transendothelial electrical resistance (TEER) measures were normalized by multiplying the individual measurements with the culture area of the permeable filter supports to achieve TEER [Ω·cm^2^] and by subtracting from the TEER measured in filter supports without cells.

The data obtained from the transport studies were plotted as total accumulated amount (mol) in the receiver chamber divided by the filter area (cm^2^) as a function of time (min), from which the steady-state fluxes were achieved as the slope of the linear plots. The apparent permeability, P_app,_ was calculated from the steady-state fluxes and the applied donor concentrations using the following equation:

1 $${P_{app}} = \frac{{{J_{ss}}}}{{{C_{donor}}}} = \frac{{{Q_t}}}{{{C_{donor}}A}}$$

J_ss_ represents the steady-state flux, C_donor_ is the initial donor concentration, Q is the transported amount, and A is the cross-sectional area of the permeable supports.

Statistical analyses were performed in Graph Pad Prism version 9 (GraphPad Software, La Jolla, CA, USA) using a two-tailed Student’s t-test or one-way ANOVA followed by Tukey’s multiple comparison test. Significance was set at *P* < 0.05. Experiments were performed by using cells from at least three individual passages (n = 3) with at least three technical replicates (N = 3) unless otherwise stated. Data are reported as mean ± standard error of the mean.

## Results

### SBAD0201-derived monolayers displayed functional barrier integrity and expression of important BBB markers at the protein level

**Fig. 1:**
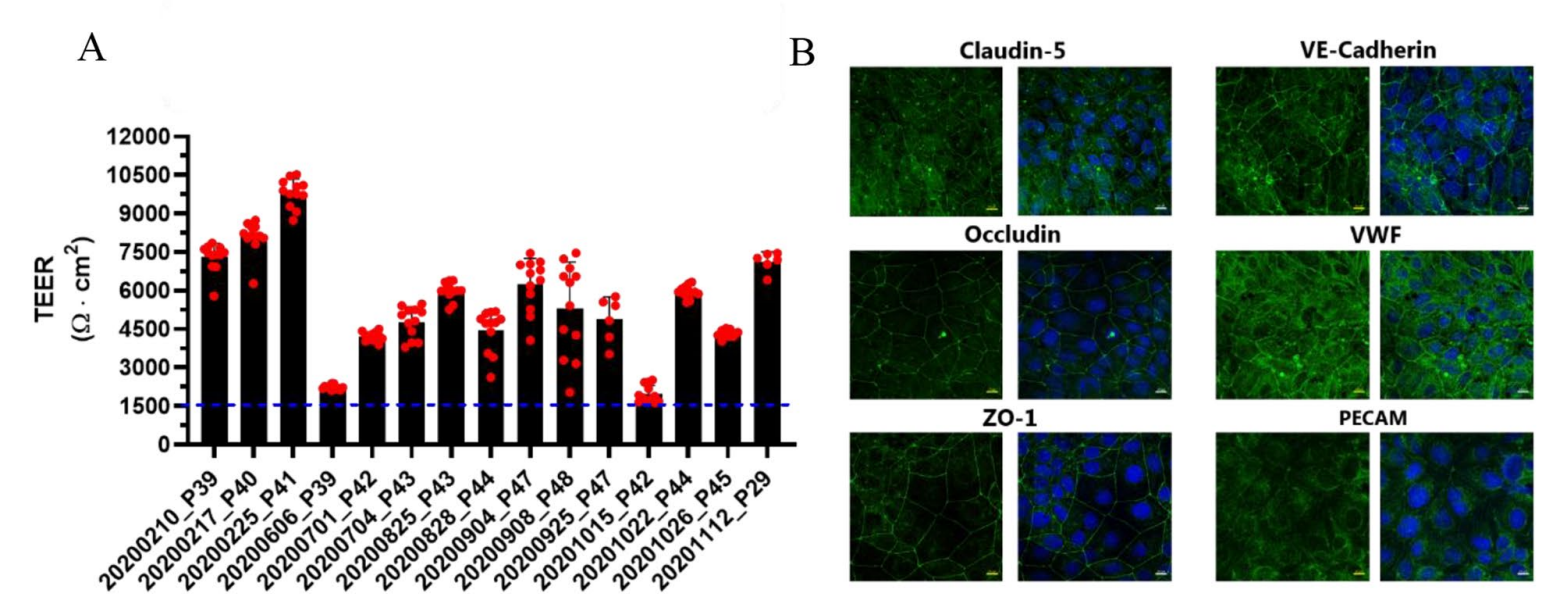
Transendothelial electrical resistance and expression of selected BBB markers in SBAD0201-derived BCECs. (**A**) Transendothelial electrical resistance across SBAD0201-derived BCECs was on Transwells in EC-/- after equilibrating to room temperature for 20 min. Data are shown as mean +/- SD of individual passage numbers (n > 6). (**B**) representative expression and localization of selected BBB tight junctions and marker proteins (green signal) by immunocytochemistry. All samples were further counterstained with DAPI (blue) to visualize cell nuclei. Images represent three individual experiments in triplicates (n = 3, total N = 9). Scale bar = 10 μm

The resulting BCECs monolayers were characterized in terms of functional tightness by measuring the electrical resistance and calculating the TEER on the experimental day (Day 10). The cell monolayers exhibited high TEER throughout the passages (Fig. 1A). Given that the TEER were found to be above 1500 Ω·cm^2^, the observed passage-dependent differences, while intriguing, do not undermine the integrity or functionality of the cell monolayers. The cell monolayers expressed claudin-5, occludin, and ZO-1 at the cell-cell contact zones, indicating proper junctional alignment (Fig. 1B). In addition, the typical endothelial markers, VE-Cadherin, VWF, and PECAM-1, were all detected at the protein level in the differentiated cells as judged by the immunostainings (Fig. 1B). VE-cadherin and VWF were found at cell-cell contact zones and in the cytosol, while PECAM-1 was localized at the contact zones and perinuclear.

**Fig. 2:**
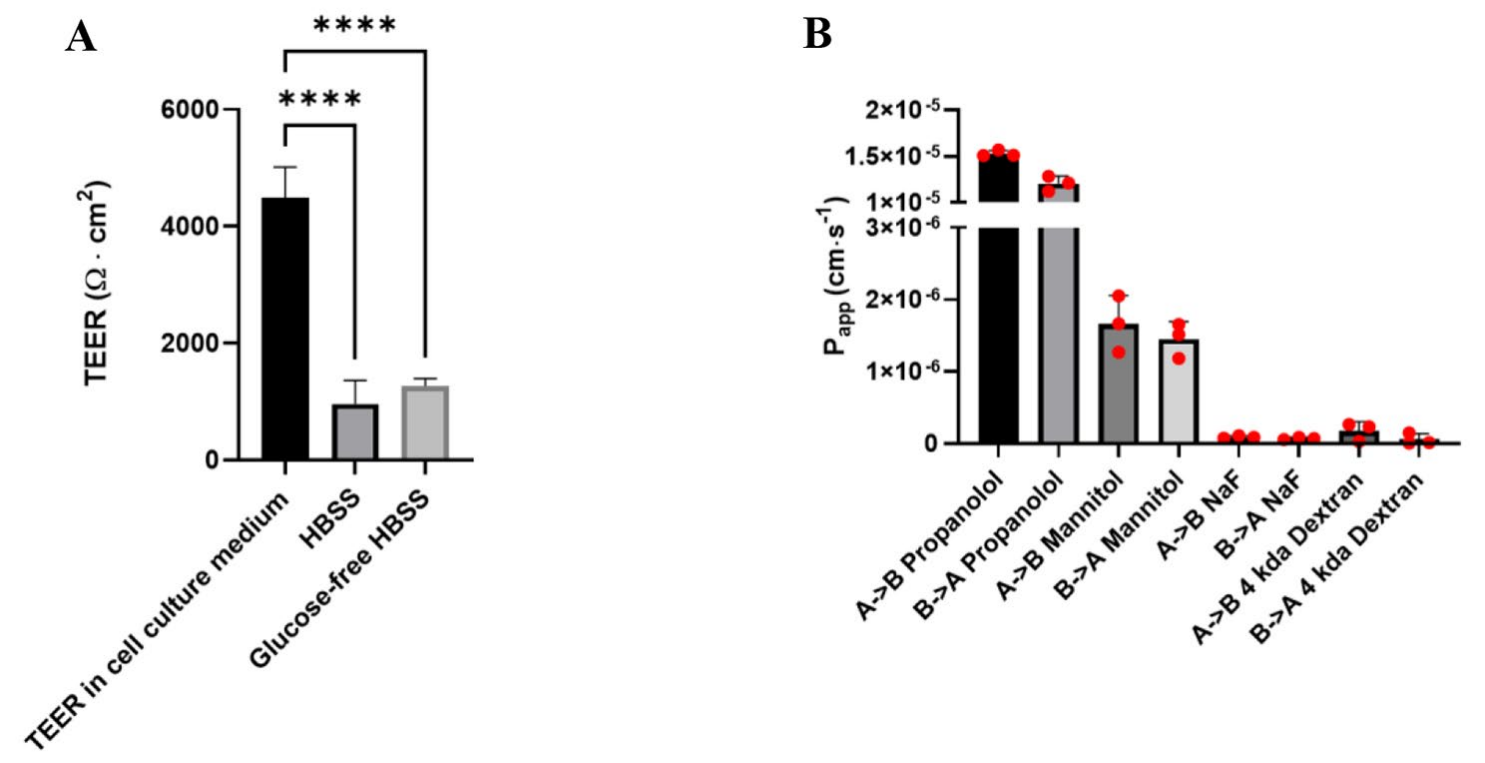
Functional barrier integrity of SBAD0201-derived BCECs. (**A**) TEER across SBAD0201-derived BCEC monolayers was investigated in EC-/- medium and upon change to transport buffer (HBSS) with and without glucose. TEER was measured on an experimental day after 20 min equilibration at room temperature on shaking table and after changing to the transport buffer (with and without glucose, 10 min equilibrium at shaking table). Data are shown as mean ± SEM of three individual passages of six replicates. Statistics: One-way ANOVA with posthoc Tukey test, ****: *p* < 0.00005. (**B**) Transport rate of paracellular and transcellular transport markers in SBAD0201-derived BCECs. Transport studies were performed over two hours for the small markers, propranolol, mannitol, and sodium fluorescein (NaF). The transport of FITC-dextran (4 kDa) was followed for four hours. Data are shown as mean ± SEM of three independent flux experiments of three technical replicates (n = 3, N = 3)

We next aimed to examine the barrier properties of BCECs derived from SBAD0201 to probe whether transport studies could be conducted in the cell model using the typical transport buffer HBSS. First, the robustness of the monolayers towards medium exchange was investigated by measuring the TEER in the conventional medium prior to medium exchange and after medium exchange to HBSS with and without glucose (Fig. 2A). The TEER in the conventional cell medium served as a control. The TEER dropped to approximately 25% of the initial TEER of 4491 ± 106 Ω·cm^2^ upon the change to HBSS with and without glucose. The monolayers exhibited a TEER of 960 ± 67 Ω·cm^2^ and 1269 ± 24 Ω·cm^2^ in HBSS with and without glucose, respectively. Still, the TEER across the monolayers in the respective transport buffer was at a level appropriate for performing transport studies and for distinguishing low- and high-permeating molecules as previously described in primary BBB models [39].

The ability of the SBAD0201-derived BCECs to distinguish between low- and high-permeating molecules was investigated by conducting bidirectional transport studies using the paracellular flux markers, [^14^C]-mannitol, sodium fluorescein (NaF), and 4 kDa FITC-dextran, and the transcellular flux marker [^3^H]-propranolol (Fig. 2B). The bidirectional transport studies on these compounds revealed that [^3^H]-propranolol displayed the highest passive permeability across the cell monolayers. The P_app_ of [^3^H]-propranolol was calculated to 1.5 ± 0.01 × 10^− 5^ cm·s^− 1^ and 1.2 ± 0.04 × 10^− 5^ cm·s^− 1^ in the apical to basolateral and basolateral to apical direction, respectively. In contrast, mannitol, NaF, and 4 kDa FITC-dextran displayed markedly lower passive-permeabilities across the cell monolayers. This demonstrates that the resulting cell monolayer is able to discriminate between low and passive-permeating molecules.

### SBAD0201-derived BCECs exhibited high functional activity of GLUT-1, LAT-1, and LRP-1, while P-gp was not functional

**Fig. 3:**
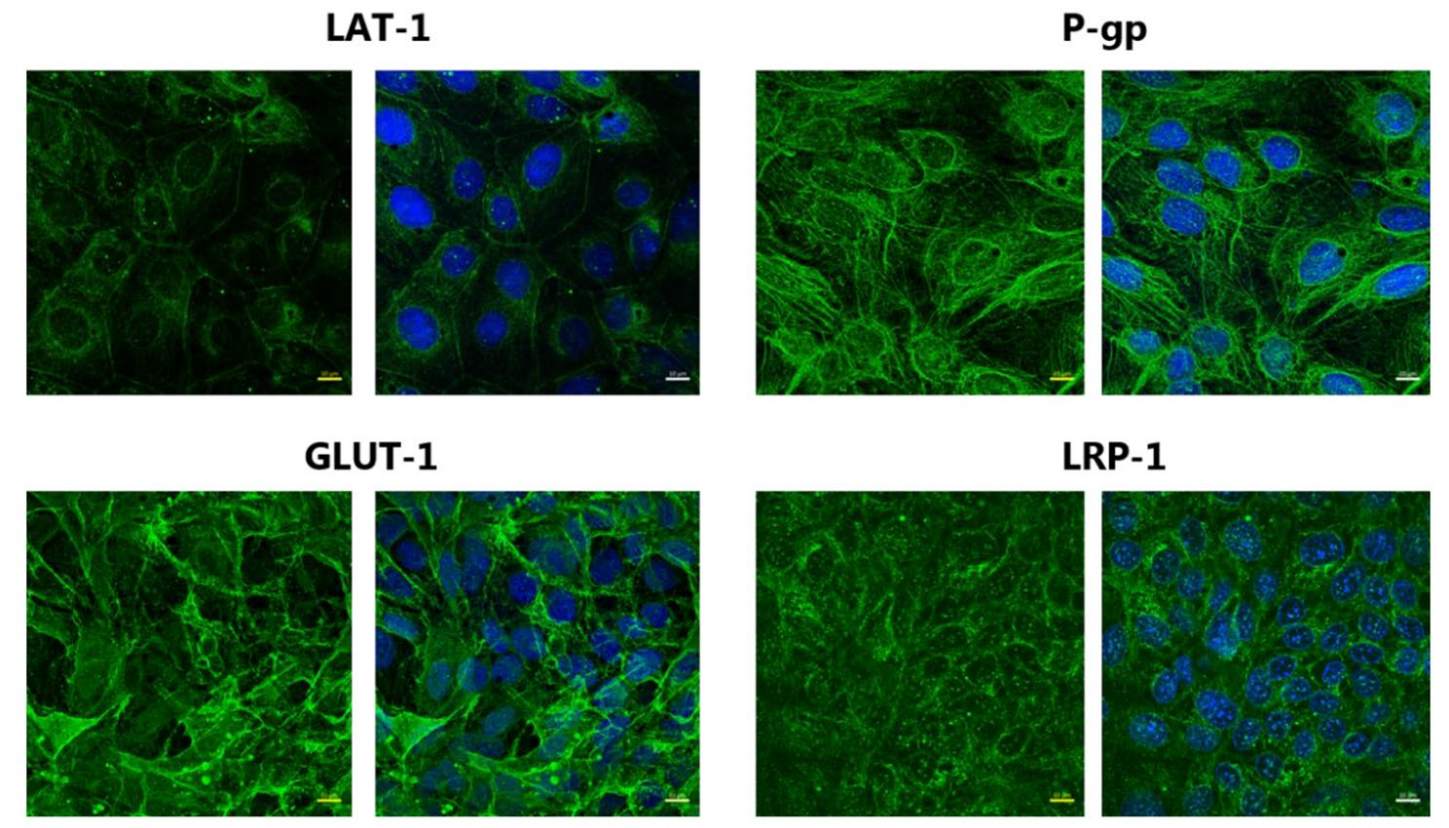
Expression of transporters and receptor in SBAD0201-derived BCECs. (**A**) Representative images of the localization of LAT-1, GLUT-1, P-gp, and LRP-1 (in green) in SBAD0201-derived BCECs (at Day 10), as determined by immunostaining. The cell nuclei were counterstained with DAPI (in blue). Images are presented as XY frontal focal planes. Scale bar = 10 μm

The transporters GLUT-1, LAT-1, P-gp, and BCRP, and the LRP-1 receptor, are all important features of the BBB and by being involved in the strict control of substance movement into and out of the brain. Therefore, the localization of the proteins was probed by immunostaining (Fig. 3).

LAT-1, GLUT-1, P-gp, and LRP-1 were all expressed as judged by the immunostaining. GLUT-1 and P-gp staining were mostly confined to the cell plasma membranes, whereas immunostaining of LAT-1 revealed signals at both the cell plasma membranes and perinuclear. LRP-1 was expressed throughout the cells, with minor staining at the cell plasma membrane.

**Fig. 4:**
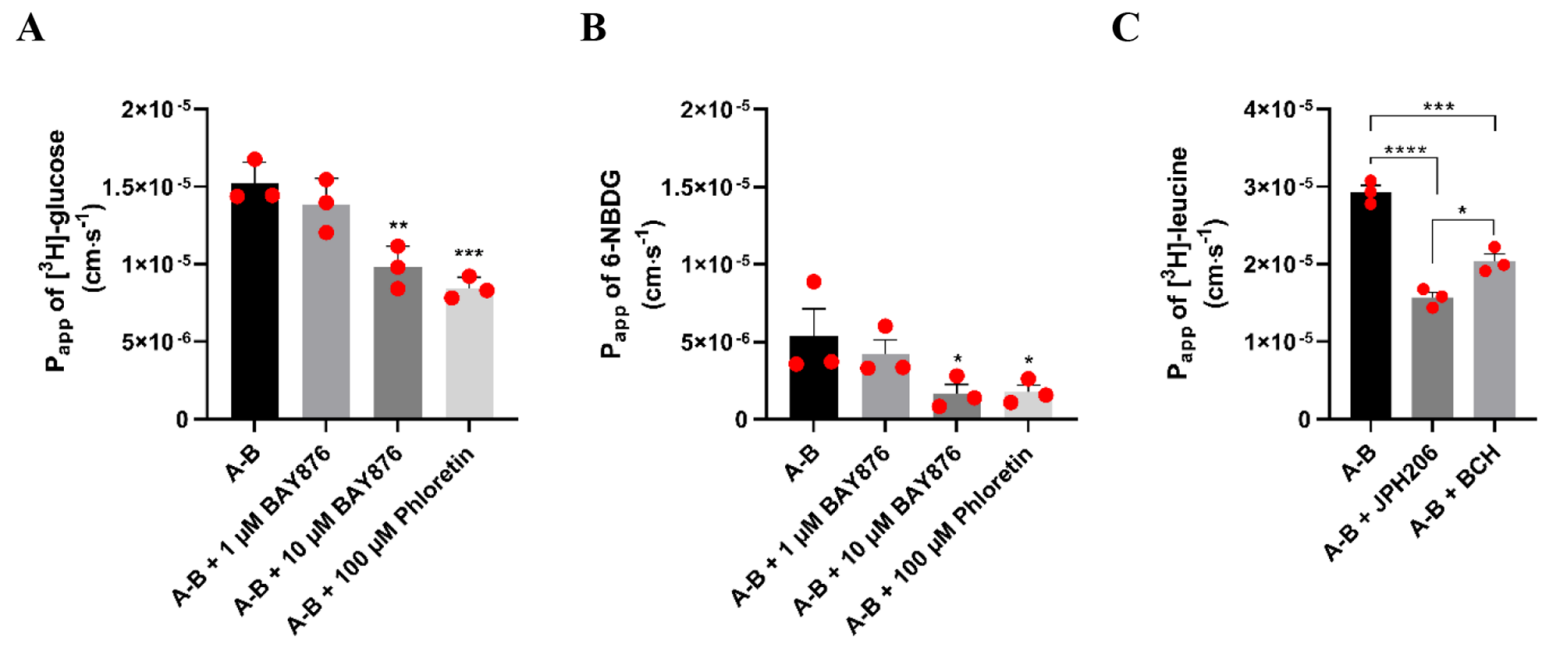
The functional activities of GLUT-1 and LAT-1. Transport of [^3^H]-glucose (20 nM) (**A**) and 6-NBDG (100 µM) (**B**) was assessed in the absence and presence of the GLUT-1 inhibitors BAY876 (1 and 10 µM) and Phloretin (100 µM). Transport of [^3^H]-L-leucine (9.3 nM) (**C**) was assessed in the absence and presence of the LAT-1 inhibitors JPH203 (10 µM) and BCH (100 µM). The transports were monitored in the apical-to-basolateral (**A**-**B**) direction over a time course of one hour. Data are shown as mean ± SEM of three individual passages of triplicates (n = 3, N = 3). Statistics: One-way ANOVA with post hoc Tukey test, *: *p* < 0.05, **: *p* < 0.005

The functional expression of the SLC-transporters, GLUT-1 and LAT-1, was investigated in SBAD0201-derived BCECs (Fig. 4). GLUT-1 activity was probed using two substrates [^3^H]-glucose (20 nM) and the fluorescent glucose analog 6-NBDG (100 µM) (Fig. 4A and B). The transport of these substrates was examined in glucose-free HBSS in apical-to-basolateral transport studies in the absence and presence of the GLUT-1 inhibitors, BAY876 and phloretin. BAY876 is a potent and highly selective inhibitor of GLUT-1 [40], exhibiting at least 130-fold selectivity for GLUT1 relative to GLUT2, GLUT3, GLUT4, whereas phloretin has a broader inhibitory specificity [41]. The apparent permeability of both [^3^H]-glucose and 6-NBDG was sensitive to the addition of 10 µM BAY876 and 100 µM phloretin (Fig. 4A and B). This indicated functional expression of GLUT-1 in the SBAD0201-derived BCECs.

LAT-1 function was investigated by a transport study in the apical-to-basolateral direction using [^3^H]-L-leucine (9.3 nM) as substrate (Fig. 4C). JPH203 and BCH were used as transport inhibitors. JPH203 represents an inhibitor of high selectivity towards LAT-1 [42], whereas BCH has a broader inhibition specificity towards multiple SLC-transporters [43]. The transport of [3H]-L-leucine was significantly decreased by co-application of JPH203 and BCH, which suggests functional expression of LAT-1.

**Fig. 5:**
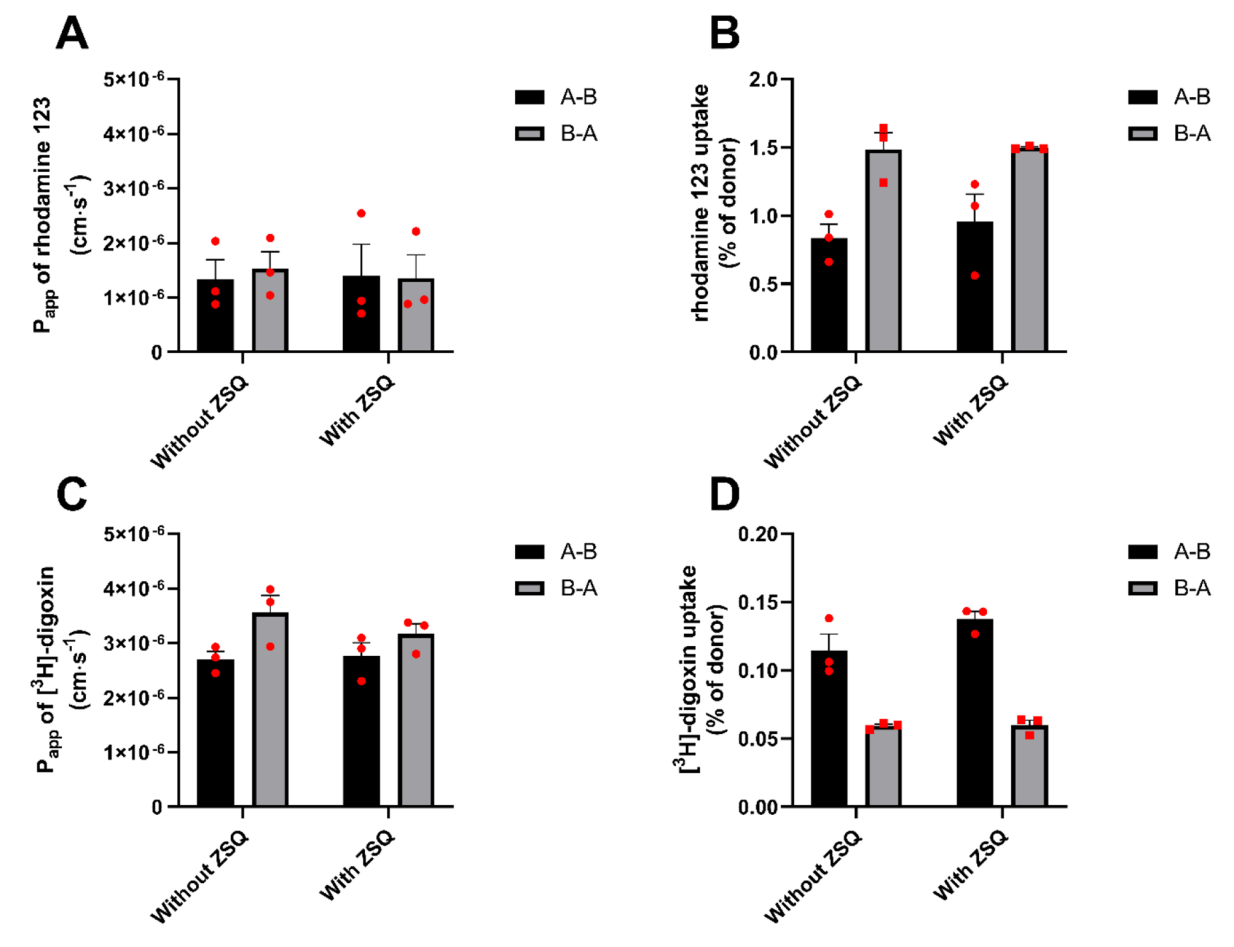
Bidirectional transport of rhodamine 123 and [^3^H]-digoxin. Transport and uptake of rhodamine 123 (5 µM) (**A** and **B**) and [^3^H]-digoxin (38.2 nM) (**C** and **D**) were assessed in the absence and presence of the P-gp inhibitor zosuquidar (ZSQ) (0.4 µM) over a time course of two hours. The apparent permeabilities were measured from the steady-state fluxes. The uptake of rhodamine 123 (**B**) and digoxin (**D**) into cells was probed after the transport study was terminated. Data are shown as mean ± SEM of three individual passages of triplicates (n = 3, N = 3)

The functional activity of P-gp in the SBAD0201-derived BCECs was examined using the well-characterized P-gp substrates digoxin and rhodamine 123. Bidirectional transport studies with [^3^H]-digoxin (38.2 nM) or rhodamine 123 (5 µM) were conducted in the absence and presence of zosuquidar (0.4 µM), which is a potent and selective P-gp inhibitor (Fig. 5).

No vectorial transport was observed for either rhodamine 123 or [^3^H]-digoxin. Furthermore, neither the transport nor the uptake of the substrates was affected by the presence of zosuquidar. This indicates that P-gp was either non-functional or extremely lowly expressed in the SBAD0201-derived BCECs, albeit P-gp expression was observed as judged by the immunostaining (Fig. 3).

**Fig. 6:**
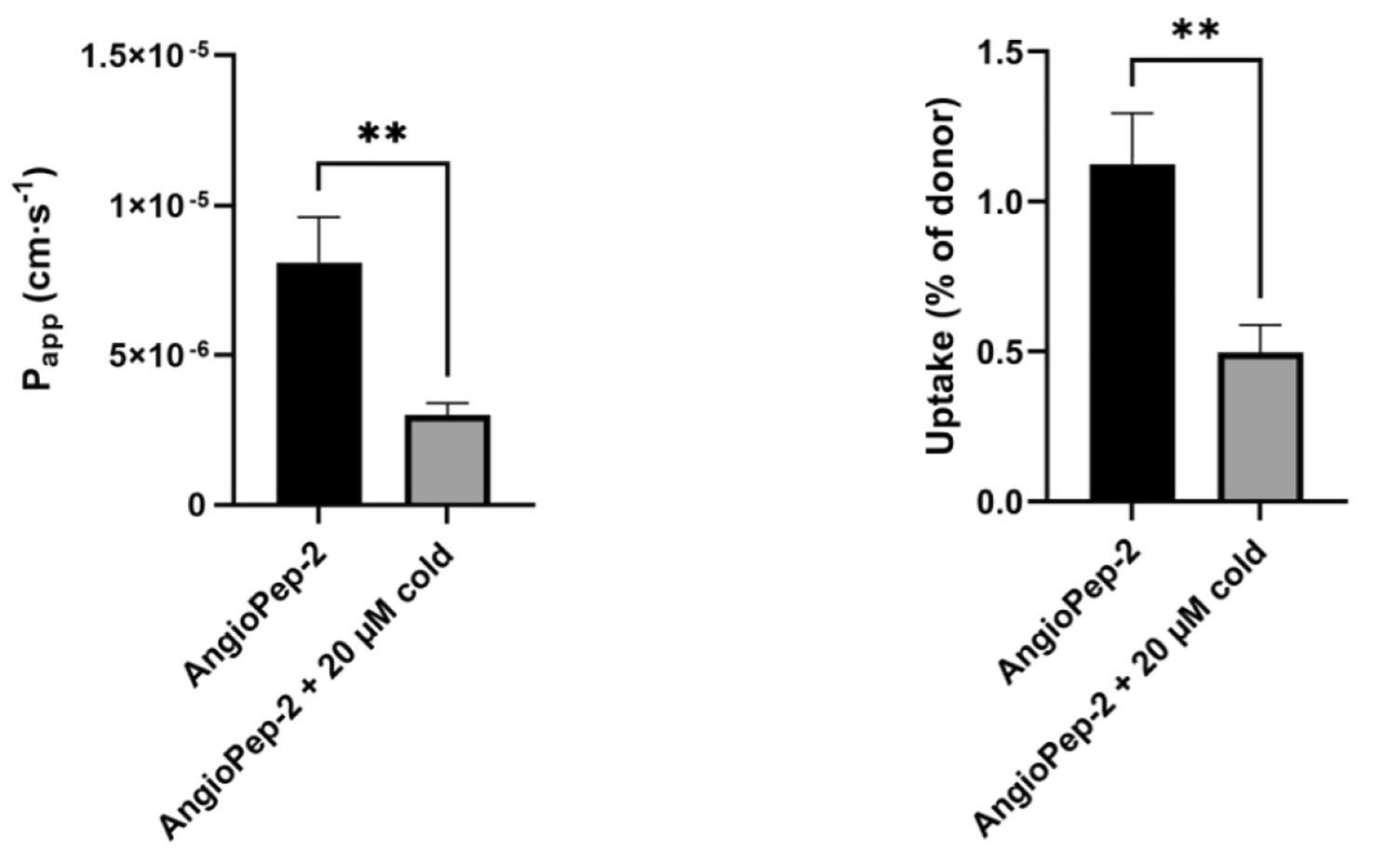
Apical to basolateral transport of [^125^I]-angiopep-2. Transport (left) and uptake (right) of [^125^I]-angiopep-2 were measured in the absence and presence of the non-labeled angiopep-2 (cold) over a time course of four hours. The cell uptake was probed after the transport study was terminated. Data are shown as mean ± SEM of three individual passages of triplicates (n = 3, N = 3). **: *p* < 0.005

BCRP function was examined in an uptake assay using mitoxantrone (20 µM) as a substrate and KO 143 as an inhibitor (Figure S1). The uptake of mitoxantrone was significantly increased by the presence of KO 143, suggesting a functional expression of BCRP in the cell model. Lastly, LRP1-mediated transcytosis was investigated by measuring the transport of [^125^I]-angiopep2 in the apical-to-basolateral direction in the absence and presence of non-labeled angiopep2 (20 µM) (Fig. 6). Both the transport (Fig. 6A) and uptake (after 4 h of transport study) (Fig. 6B) were sensitive to the addition of non-labeled angiopep2, suggesting functional activity of LRP-1 in the SBAD0201-derived BCECs.

### SLC-transporters and receptors showed higher expression levels than efflux transporters, as judged by transcriptomics and proteomics

**Fig. 7:**
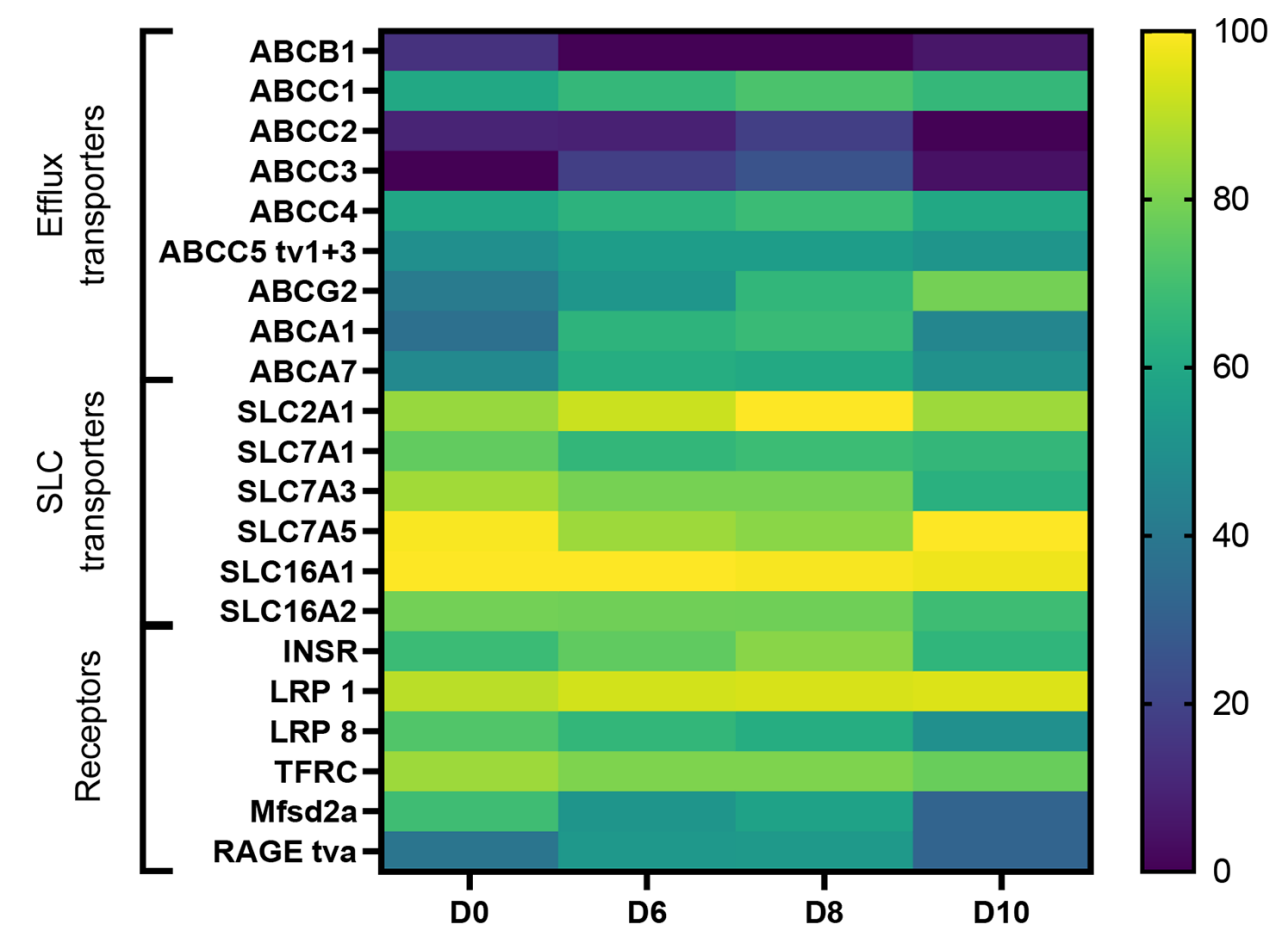
Heat map of mRNA transcript levels of selected transporter and receptor genes in SBAD0201-derived cells during differentiation. RNA samples were isolated from the cells on Day 0 (D0), Day 6 (D6), Day 8 (D8), and Day 10 (D10). Data were normalized relative to the reference genes, GAPDH and B-actin. The color code legends were formatted in log-scale and normalized to the lowest value (ABCB1, D6). Yellow represents the highest mRNA expression, and dark blue has low mRNA expression. tv: transcript variant

To delve deeper into the intricacies of the cell model, particularly in light of the absence of functional P-gp, we conducted transcriptomic and proteomic studies to thoroughly characterize the cell model. The mRNA expression levels of selected SLC transporters, efflux transporters, and receptors were investigated during the differentiation period (Fig. 7). At Days 0, 6, 8 and 10, cells were lysed during the stem cell differentiation, and the RNA content was isolated to follow the apparent transcript profiles. For this purpose, a high-throughput qPCR chip developed by AIT [27, 44, 45] was used to probe the changes during SBAD0201 differentiation and to gain insight into levels of the investigated genes.

Among the efflux-transporters, ABCG2 (BCRP), ABCC1 (MRP1), and ABCC4 (MRP4) displayed the highest expression levels, whereas ABCB1 (P-gp) and ABCC2 (MRP2), which are both described as being involved in the BBB function, were among the lowest expressed throughout the differentiation period. SLC2A1 (GLUT-1), SLC7A5 (LAT-1), and SLC16A1 (MCT-1) were expressed in high abundance, as judged by the relative expression levels. Concerning receptors, transcript levels for LRP1 were found in the highest abundance when compared to TFRC and INSR. TFRC and INSR, previously described as the most expressed receptors at the BBB [46], were detected at the mRNA level. Similar to tight junctions and adherens junctions, the expression levels of transporter and receptor genes were upregulated from Day 0 to Day 8, followed by a decrease on Day 10. This pattern did, however, not apply to ABCG2 and SLC7A5. While the expression of ABCG2 was progressively increasing during the culture, SLC7A5 expression gradually decreased from Day 0 to Day 8, followed by a marked increase on Day 10 to the level observed at Day 0.

Furthermore, the protein expression amounts of four ABC transporters and six SLC transporters were examined in the SBAD0201-derived cells on Day 0 and Day 10 and compared to those estimated in hCMEC/D3 cells using LC-MS/MS -based quantitative targeted absolute proteomics (Table 2). BCRP was the most abundantly expressed among the efflux transporters in SBAD0201-derived BCECs and hCMEC/D3 cells, followed by MRP1. The protein amounts of P-gp were below the limit of quantification in the SBAD0201-derived cell types, suggesting a low expression of P-gp at both Day 0 and Day 10. P-gp was however found at a level comparable to BCRP expression in hCMEC/D3 cells. Among the SLC-transporters, GLUT-1 was the most highly expressed, whereas LAT-1 and MCT-1 were among the lowest expressed. FATP1, both expressed in brain capillary endothelium [47, 48], were not detected in SBAD0201-derived cells at D0 and BCECs, while it was found to be expressed at a level in hCMEC/D3 cells.

**Table 2:** Protein expression amounts of SLC and ABC transporters in crude membrane fractions of SBAD0201 cells (n = 8–9 biological replicates) prior to differentiation (D0) and after complete differentiation (D10) and in hCMEC/D3 cells (n = 7 biological replicates)

Transporter	SBAD0201 cells at D0 fmol/µg total protein mean ± SD	SBAD0201-derived BCECs at D10 fmol/µg total protein mean ± SD	hCMEC/D3 cells fmol/µg total protein mean ± SD
ABCB1/P-gp	< ULQ	< ULQ	0.24 ± 0.05
ABCG2/BCRP	< ULQ	0.18 ± 0.094	0.27 ± 0.13
ABCC1/MRP1	0.18 ± 0.066	0.11 ± 0.048	0.21 ± 0.07
ABCC4/MRP4	0.038 ± 0.016	0.021 ± 0.0097	0.026 ± 0.0088
SLC2A1/GLUT1	9.7 ± 2.2	6.9 ± 2.7	3.0 ± 1.0
SLC7A1/CAT1	1.2 ± 0.29	0.27 ± 0.069	0.36 ± 0.11
SLC7A5/LAT1	0.29 ± 0.14	0.11 ± 0.034	0.14 ± 0.07
SLC3A2/4F2hc	1.3 ± 0.65	0.53 ± 0.21	0.18 ± 0.085
SLC16A1/MCT1	0.23 ± 0.19	0.11 ± 0.073	0.040 ± 0.024
SLC27A1/FATP1	< ULQ	< ULQ	0.048 ± 0.024

ULQ – under limit of quantification. The protein expression was considered below the limit of quantification (LOQ) if signal peaks were obtained for only two or one MRM transition(s). The LOQ was estimated as the lowest concentration of a stable isotope-labelled peptide (spiked into the sample containing 50 µg of total protein followed by processing similarly as the study samples) for which signal peaks were achieved by three or more transitions

### The SBAD0201-derived cells expressed a wide range of different tight junctions and adherens junctions during differentiation

**Fig. 8:**
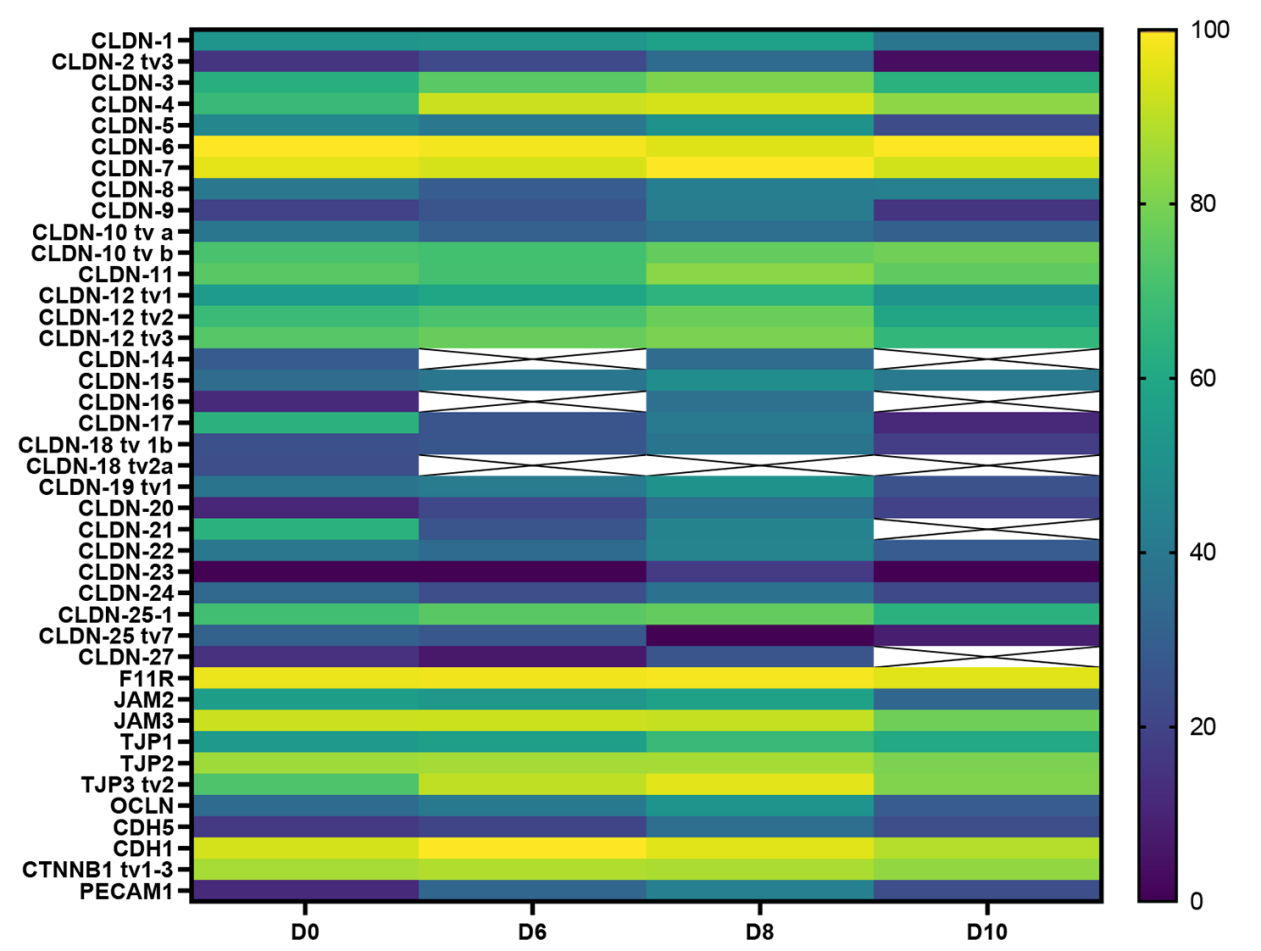
Heat map of mRNA transcript levels of tight junctions and adherens junctions in SBAD0201-derived cells during differentiation. RNA samples were isolated from the cells on Day 0 (D0), Day 6 (D6), Day 8 (D8), and Day 10 (D10). Data were normalized relative to the reference genes, GAPDH and ACTB. The color code legends were formatted in log-scale and normalized to the lowest value (CLDN23, D0). Yellow represents the highest mRNA expression, and dark blue low mRNA expression. tv: transcript variant

The mRNA expression levels of selected SLC transporters, efflux transporters, and receptors were also investigated by the high-throughput qPCR chip during the differentiation period at Day 0, 6, 8 and 10 (Fig. 8).

CLDN-5, OCLN, TJP1 (ZO-1), PECAM-1, and CDH5 were all detected throughout the culture period. However, CLDN-4, -6, and − 7 were found in markedly higher abundance throughout the differentiation period than the mRNA of the other CLDNs. Interestingly, the CLDN5 transcript levels were relatively low and among the lowest expressed TJs and AJs genes, even after full differentiation (D10). CLDN-1, -3, -8, -11, -12, and − 25 exhibited moderate transcript levels. The transcript of VE-cadherin (CDH5) and PECAM-1 seemed relatively low in the SBAD0201 cells throughout the differentiation period. However, they displayed higher expression on Day 10 compared to Day 0. On the other hand, CDH1, an epithelial cell marker, was highly expressed throughout the differentiation period. Concerning the zonula occludens encoding genes TJPs, TJP1 exhibited the lowest expression compared to TJP2 and TJP3. Besides the TJPs and claudins, the JAMs, although not essential for TJ formation in endothelial or epithelial cells [49], were expressed throughout the differentiation period, with JAM1 being the most expressed. Interestingly, the expression levels of the investigated tight junctions and adherens junctions gradually increased from Day 0 to Day 8, followed by a down-regulation on Day 10. This indicates that the final seeding on permeable supports may cause a decrease in the mRNA expression of the junctional proteins.

## Discussion

Within the last decade, BBB models based on hiPSCs have been widely applied as a robust tool for investigating different aspects of the BBB. The BBB models based on hiPSCs or other stem cell sources can benefit early-phase drug permeability studies during drug development [24]. An overarching advantage of the hiPSCs, when compared to other in vitro BBB models, is their human origin, which may improve the in vitro to human translation. However, using hiPSC-derived models has recently been questioned for BBB studies [50]. The differentiated BCECs have been shown to exhibit epithelial traits, such as expression of the epithelial cellular adhesion molecule (EPCAM) and E-cadherin along with other epithelial markers, low expression of claudin-5, and questionable brain capillary endothelial cell morphology [50]. The present study aimed at establishing and characterizing a hiPSC-derived BBB model using the SBAD0201 cell line. This was done to evaluate the applicability and robustness of the SBAD0201-derived BCECs for investigations on the BBB. The hiPSC differentiation protocol was established with minor modifications to the protocol described by Lippmann et al. [16, 17]. We conducted a transcriptomic study using a high-throughput qPCR approach to examine the development of the transcript levels of various tight junctions, adherens junctions, transporters, and receptor systems during culture. It was observed that CLDN5 was expressed at very low mRNA amounts. This was in agreement with the study by Lu et al., which similarly showed that hiPSC-derived brain capillary endothelial-like cells using the IMR90 cell line exhibited low expression levels of CLDN5 [50]. On the other hand, CLDN4, -6, and − 7 were expressed in the highest abundance when compared to the other claudins throughout the differentiation period. CLDN4, -6, and − 7 have previously been described as predominantly expressed in epithelial barriers rather than an endothelial barrier [51–54]. In support, a transcriptomic study by Zhang et al. showed relatively low transcript levels of CLDN4, -6, and − 7 in human brain capillaries [55]. 

Several studies on the BBB have emphasized the important role of claudin-5 as one of the most expressed tight junctions in the brain capillary endothelium [56–58]. Indeed, a recent study proposed CLDN5, -11, -12 and − 25 as the highest expressed CLDNs in human brain capillaries, while CLDN4 and − 6 transcripts were lower than the transcript of CLDN5 [59]. In contrast CLDN5, CLDN11, -12, and − 25 transcript levels were moderate in the SBAD0201-derived cell monolayers, as judged by the qPCR data. However, the high expression of the epithelial claudin gene repertoire (CLDN4, -6, and − 7) and low expression of P-gp suggest that the SBAD0201-derived BCECs still display some immaturity and are not fully differentiated, which is also reported by Singh et al. [60]. This also applies to other BBB models based on hIPSC cell lines [16–22]. This is further supported by the relatively low transcript levels of PECAM-1 and CDH5 (VE-cadherin), even though both PECAM-1 and VE-cadherin were found to be expressed and localized at the junctional zones in the SBAD0201-derived BCECs. E-cadherin is a well-known adherens junction important for forming tight junctions in choroid plexus epithelial cells and its presence has also been detected at the mouse BBB [61]. It should be noted that transcript levels do not necessarily correlate with protein levels due to the complex regulation of gene expression [62]. It should also be noted that immunostaining of claudin-5 demonstrated correct localization at the cell–cell contacts, albeit low transcripts levels were observed. Still, the SBAD0201-derived BCECs were capable of forming extremely tight monolayers with TEER of 5474 ± 167 Ω·cm^2^ higher than the in vivo BBB estimated TEER (1200–1900 Ω·cm^2^) [63, 64], as well as those estimated in other hiPSC-derived BBB models (> 1400 Ω·cm^2^) [16, 18, 65–69]. Furthermore, the SBAD0201-derived BCECs also demonstrated the capability to distinguish between low- and high-permeating molecules to the same degree as other hiPSC-derived BBB models, as shown in the permeabilities of the transcellular and paracellular flux markers. This suggests that the model established in the present study is suited for studying passive BBB permeability. The efflux transporters constitute a major part of the BBB restrictive function. In contrast, molecule affinity for the efflux transporters, P-gp or BCRP, is a major determinant for drug candidate selection during industrial drug development [70]. Interestingly, the efflux transporters were found at relatively low mRNA levels in BCECs compared during the entire differentiation period except for the gene encoding for BCRP. BCRP has been suggested to be the main and highest expressed efflux transporter at the human BBB by some studies [33, 38]. We similarly found BCRP as the highest expressed efflux transporter among the investigated efflux transporter, while the expression level of P-gp was under the limit of quantification at absolute protein levels. Interestingly, Uchida et al. previously demonstrated that the expression of MRP1 was under the limit of quantification in human brain capillaries (Uchida et al., 2011). In contrast, it exhibited a relatively high expression in SBAD0201-derived BCECs. The presence of MRP1 at the BBB remains controversial [55, 71]. In the present study, P-gp was functionally inactive in the SBAD0201-derived cell monolayers, as judged by the transport assays using the P-gp substrates [^3^H]-digoxin and rhodamine 123 and the P-gp inhibitor, zosuquidar. This observation agreed with our earlier study on the Bioni010-C hiPSC line-derived BCECs, which did not display functional P-gp or BCRP [18]. Other studies have demonstrated functional activities of P-gp along with other efflux transporters in the hiPSC-derived BBB models in transport assays as well as in uptake assays [16, 65–67, 69, 72]. However, Ohshima et al. also observed that P-gp expression in their hiPSC-derived BBB model was insufficient for examining interactions between drug compounds and P-gp [68]. Thus, there are conflicting results regarding the functionality of P-gp in the hiPSC-derived BBB models, which in fact could be due to differences in protocols, different hiPSC lines and handling during culture and differentiation. Thus, in vitro BBB models based on primary cell sources, in which efflux transporters have been extensively characterized, may be more suited for such purposes. On the other hand, primary endothelial cells are often reported to display low functional expression of the SLC-transporters, thereby making them insufficient for studying such these [7]. In contrast, the hiPSC-derived BBB models have been reported to express SLC transporters in high abundance [16, 18, 65–69]. This was also reflected in the present study, where the SBAD0201-derived BCECs exhibited expression of SLC transporter and receptor genes in high abundance. The high expression of SLC transporters could further be confirmed at absolute protein levels.

The transport of [^125^I]-angiopep-2, sensitive to the addition of non-labelled angiopep-2, indicated functional LRP-1 in the BCECs derived from SBAD0201 cells. The P_app_ of [^125^I]-angiopep-2 was 8.1 ± 2.9 × 10^− 6^ cm·s^− 1^ in the absence of non-labelled angiopep2. This value is consistent to the P_app_ estimated in another hIPSC cell line-derived (Bioni010C) BCECs (9.7 ± 3.9 × 10^− 6^ cm·s^− 1^) [18], and lower than the angiopep2 P_app_ estimated in an uptake assay using in a rat brain endothelial cell line RBE-4 (~ 4.4 cm·s^− 1^, calculated from Fig. 3 in [73]). The observed high P_app_ of angiopep-2 through SBAD0201-derived BCECs might be a result of the high activity of LRP-1 in the hIPSC-derived BCECs. The use of the pulse-chase method to investigate intracellular uptake of angiopep2 will provide more insights into the transport dynamics of angiopep-2 in the SBAD0201-derived BCECs.

Overall, GLUT-1, LAT-1, BCRP, and LRP-1 functionalities in the model were verified in the transport and uptake assays. This highlights the utility of this model as a screening tool for passive permeability ranking and investigations on GLUT-1, LAT-1, BCRP, and LRP-1 mediated transport across the BBB.

## Conclusions

The SBAD0201-derived BCECs exhibited an extremely tight barrier with an expression of claudin-5, ZO-1, and occludin at the cell-cell contacts. The cell monolayers displayed the ability to distinguish between low- and high permeating molecules. The model demonstrated expression of GLUT-1, LAT-1, and LRP-1, as well as functional transcellular transport of model substrates, and may therefore be applicable to examine these transport pathways across the endothelial cell monolayer. Expression of several important BBB markers, such VE-cadherin, PECAM1, and VWF was confirmed. However, the full identity of the model has yet to be clarified, as the cells shown to encompass high transcript level of the gene encoding for E-cadherin and low transcript of CLDN-5. Even though P-gp seemed to be expressed in the SBAD0201-derived BCECs, P-gp was not detected when quantifying absolute protein levels, and was found to be functionally inactive. The SBAD0201-derived BCECs are thus suitable for examining passive BBB permeability, functional analysis of GLUT-1, LAT1, BCRP and LRP-1 in drug development studies and potentially for MCT-1 as well but lacks functional expression of P-gp.

### Electronic supplementary material

Below is the link to the electronic supplementary material.

Supplementary Material 1

## Data Availability

The datasets used and/or analyzed during the current study are available from the corresponding author on request.
