# Supplementary material for: Characterization of an iPSC-based barrier model for blood-brain barrier investigations using the SBAD0201 stem cell line
Source: Fluids Barriers CNS. 2023 Dec 19;20:96. doi: 10.1186/s12987-023-00501-9 (PMC10731806; doi:10.1186/s12987-023-00501-9)
Supplement: Supplementary file 1 — Supplementary Material 1 [file 12987_2023_501_MOESM1_ESM.docx]

# Supplementary Material for: Characterization of an iPSC-based barrier model for blood-brain barrier investigations using the SBAD0201 stem cell line

# Authors;

Burak Ozgür^1,2^, Elena Puris^3^, Andreas Brachner^4^, Antje Appelt-Menzel^5,6^, Sabrina Oerter^5,6^, Viktor Balzer^3^, Mikkel Roland Holst^7^, Rasmus Folmann Christiansen^2^, Kathrine Hyldig^2,7^, Stephen T. Buckley^8^, Mie Kristensen^1^, Seppo Auriola^9^, Allan Jensen^2^, Gert Fricker^3^, Morten Schallburg Nielsen^7^, Winfried Neuhaus^4,10^ & Birger Brodin^1^

1. Department of Pharmacy, University of Copenhagen, Universitetsparken 2, DK-2100, Copenhagen, Denmark
2. Biotherapeutic Discovery, H. Lundbeck A/S, DK-2500, Valby, Denmark
3. Institute of Pharmacy and Molecular Biotechnology, Ruprecht-Karls-University, Heidelberg, Germany
4. AIT - Austrian Institute of Technology GmbH, 1210 Vienna, Austria
5. University Hospital Würzburg, Chair Tissue Engineering and Regenerative Medicine (TERM), 97070 Würzburg, Germany
6. Fraunhofer Institute for Silicate Research ISC, Translational Center Regenerative Therapies (TLC-RT) Röntgenring 11, 97070 Würzburg, Germany
7. Department of Biomedicine, Aarhus University, DK-8000, Aarhus, Denmark
8. Global Research Technologies, Novo Nordisk A/S, DK-2760, Måløv, Denmark
9. School of Pharmacy, University of Eastern Finland, Kuopio, Finland
10. Department of Medicine, Faculty of Medicine and Dentistry, Danube Private University, 3500 Krems, Austria

*Corresponding author:

Birger Brodin, Department of Pharmacy, University of Copenhagen, Universitetsparken 2, DK-2100, Copenhagen, Denmark Email; [birger.brodin@sund.ku.dk](mailto:birger.brodin@sund.ku.dk)

Voice: +4535336166

**Supplementary Table S1.** *Probe peptide amino acid sequences and multiple reaction monitoring (MRM) transitions for the LC-MS/MS-based quantitative targeted absolute proteomics analysis.*

| **Gene/Protein name** | **St/IS** | **Unique amino acid sequence** | **Retention time (min)** | **MRM transitions (m/z)** | | | | |
| --- | --- | --- | --- | --- | --- | --- | --- | --- |
|  |  |  |  | **Q1** | **Q3.1** | **Q3.2** | **Q3.3** | **Q3.4** |
| **ABC transporters** | | | | | | | | |
| **ABCB1/P-gp** | St | NTTGALTTR | 8.7 | 467.7 | 719.4 | 618.3 | 561.3 |  |
|  | IS | NTTGALTT**R*** | 8.7 | 472.7 | 729.4 | 628.3 | 517.3 |  |
| **ABCG2/BCRP** | St | SSLLDVLAAR | 27.7 | 522.8 | 757.4 | 644.3 | 529.3 |  |
|  | IS | SSLLDVLAA**R*** | 27.7 | 527.8 | 767.4 | 654.3 | 539.3 |  |
| **ABCC1/MRP1** | St | TPSGNLVNR | 9.7 | 479.2 | 759.4 | 672.3 | 501.3 |  |
|  | IS | TPSGNLVN**R*** | 9.7 | 484.2 | 769.4 | 682.3 | 511.3 |  |
| **ABCC4/MRP4** | St | APVLFFDR | 24.8 | 482.7 | 796.4 | 697.3 | 584.2 |  |
|  | IS | APVLFFD**R*** | 24.8 | 487.7 | 806.4 | 707.3 | 594.2 |  |
| **SLC transporters** | | | | | | | | |
| **SLC2A1/GLUT1** | St | TFDEIASGFR | 21.4 | 571.7 | 894.4 | 779.4 | 650.4 | 537.3 |
|  | IS | TFDEIASGF**R*** | 21.4 | 576.7 | 904.4 | 789.4 | 660.4 | 547.3 |
| **SLC3A2/4F2hc** | St | VAGSPGWVR | 14.6 | 464.7 | 829.4 | 758.4 | 701.4 | 614.3 |
|  | IS | VAGSPGWV**R*** | 14.6 | 469.7 | 839.4 | 768.4 | 711.4 | 624.3 |
| **SLC7A1/CAT1** | St | TILSPK | 11.7 | 329.7 | 557.4 | 444.4 | 331.2 |  |
|  | IS | TILSP**K*** | 11.7 | 333.7 | 565.4 | 452.4 | 339.2 |  |
| **SLC7A5/LAT1** | St | VQDAFAAAK | 12.1 | 460.7 | 821.4 | 578.3 | 507.3 |  |
|  | IS | VQDAFAAA**K*** | 12.1 | 464.8 | 829.4 | 586.3 | 515.3 |  |
| **SLC16A1/MCT1** | St | SITVFFK | 23.5 | 421.3 | 641.4 | 441.2 | 294.2 |  |
|  | IS | SITVFF**K*** | 23.5 | 425.3 | 649.4 | 449.2 | 302.2 |  |
| **SLCO1C1/**  **OATP1C1** | St | DFLPSLK | 22.8 | 410.2 | 557.4 | 444.3 | 263.1 |  |
|  | IS | DFLPSL**K*** | 22.8 | 414.2 | 565.4 | 452.3 | 263.1 |  |
| **SLC27A1/FATP1** | St | LLPQVDTTGTFK | 20.5 | 660.4 | 1093.6 | 996.5 | 868.44 | 769.4 |
|  | IS | LLPQVDTTGTF**K*** | 20.5 | 664.4 | 1101.6 | 1004.5 | 876.4 | 777.4 |

St – standard, IS – internal standard

Bold letter with* denotes labelled arginine (R) or lysine (K) with a stable isotope ^13^C and ^15^N


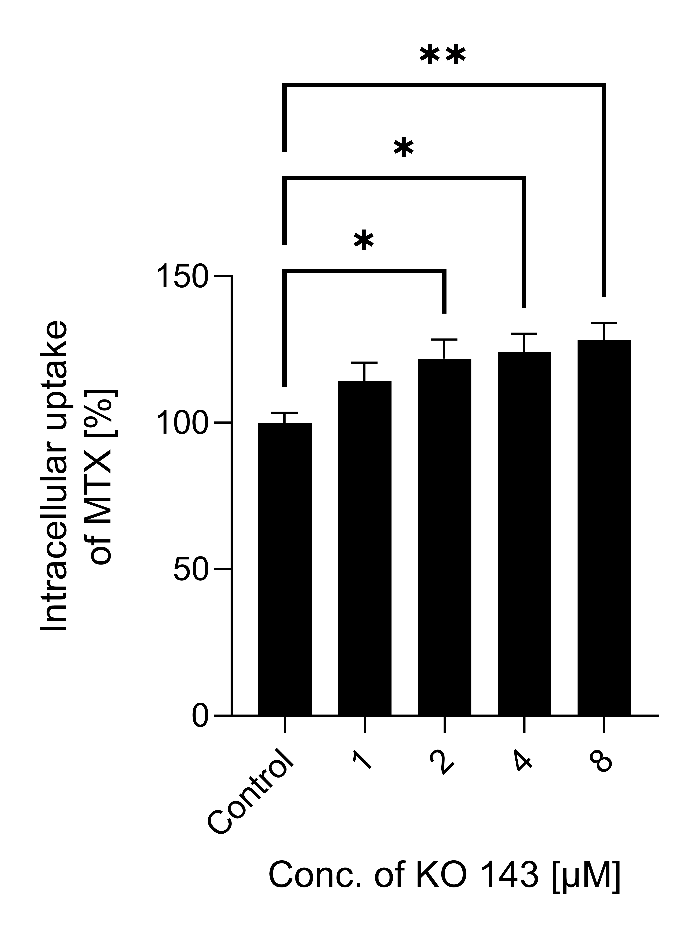


Figure S1. Concentration-dependent effects of the BCRP inhibitor KO 143 on the cellular accumulation of mitoxantrone (MTX) in SBAD0201 derived BCECs. Cells were treated with KO 143 in concentrations ranging from 1 to 8 µM for 4 h, followed by 1 h incubation with 20 µM of MTX. Increase of intracellular fluorescence was measured by a plate reader Infinite® P200 Pro with excitation and emission wavelengths of 610 nm and 685 nm. All measured fluorescence values were corrected by subtracting the background fluorescence, and normalized towards the values determined in the control group. Results are shown as mean ± SD, n = 3, N = 3. Statistical significance was determined using one-way analysis of variance (ANOVA) followed by Dunnett's post hoc test. Differences were considered significant at p*< 0.05, p** < 0.005.
